# Administration of neostigmine after tracheal intubation shortens time to successful intraoperative neuromonitoring during thyroid surgery: a randomized controlled trial

**DOI:** 10.1038/s41598-022-21282-5

**Published:** 2022-10-07

**Authors:** Moon Young Oh, Young Jun Chai, Tzu-Yen Huang, Che-Wei Wu, Gianlorenzo Dionigi, Hoon Yub Kim, Chanho Kim, Dongwook Won, Jung-Man Lee

**Affiliations:** 1 Department of Surgery, Seoul National University College of Medicine, Seoul, Republic of Korea; 2 Department of Surgery, Seoul National University College of Medicine, Seoul Metropolitan Government - Seoul National University Boramae Medical Center, Seoul, Republic of Korea; 3 Transdisciplinary Department of Medicine and Advanced Technology, Seoul National University Hospital, Seoul, Republic of Korea; 4 Department of Otorhinolaryngology–Head and Neck Surgery, Kaohsiung Medical University Hospital, Kaohsiung Medical University, Kaohsiung, Taiwan; 5 Department of Pathophysiology and Transplantation, University of Milan, Milan, Italy; 6 Division of Surgery, Istituto Auxologico Italiano Instituto di Ricovero e Cura a Carattere Scientifico (IRCCS), Milan, Italy; 7 Department of Surgery, KUMC Thyroid Center, Korea University Anam Hospital, Korea University College of Medicine, Seoul, Republic of Korea; 8 Department of Anesthesiology and Pain Medicine, Seoul National University Hospital, Seoul, Republic of Korea; 9 Department of Anesthesiology and Pain Medicine, Seoul National University College of Medicine, Seoul Metropolitan Government - Seoul National University Boramae Medical Center, 20, Boramae-ro 5-gil, Dongjak-gu, Seoul, 07061 Republic of Korea

**Keywords:** Endocrinology, Medical research

## Abstract

This prospective, randomized controlled trial evaluated the effect of neostigmine for intraoperative neuromonitoring (IONM) during thyroid surgery. Forty subjects undergoing thyroidectomy with IONM, randomized into neostigmine administration after tracheal intubation (Group N, n = 20) or control treatment with normal saline (Group C, n = 20), completed the trial. Electromyography amplitudes of the vagus nerve (V1) were recorded before thyroid dissection. The time from the initial V1 signal check to successful V1 stimulation was recorded. In Group N, all the patients had a successful V1 signal at the first check, whereas ten (50%) patients in Group C had a time delay between the initial V1 check and successful V1 (*p* < 0.001). The mean delay time among the delayed patients in Group C was 11.2 ± 1.4 min. The mean time from skin incision to successful V1 stimulation was significantly shorter in Group N than in Group C (15.4 ± 2.4 min vs. 19.9 ± 5.7 min, *p* = 0.003). In Groups N and C, the mean V1 amplitudes were 962.2 ± 434.5 μV vs. 802.3 ± 382.7 μV (*p* = 0.225), respectively, and the mean R1 amplitudes were 1240.0 ± 836.5 μV vs. 1023.4 ± 455.8 μV (*p* = 0.316), respectively. There was one bucking event in Group N. In conclusion, neostigmine administration immediately after tracheal intubation can be useful to reverse neuromuscular blockade for successful IONM in thyroid surgeries.

## Introduction

The use of intraoperative neuromonitoring (IONM) has increased in thyroid surgery because such monitoring has been shown to reduce recurrent laryngeal nerve (RLN) injury with its use^1^. Accordingly, interest in improving the quality of IONM is growing. One of the key factors for successful IONM is the adequate reversal of neuromuscular blockade. Although neuromuscular blocking agents (NMBAs) are needed to optimize tracheal intubation conditions and operating conditions, excessive neuromuscular blockade may interfere with electromyography (EMG) signals during IONM^2^.

Sugammadex, a rapid-acting NMBA, is considered to be the most effective reversal agent for IONM to date^3^, but its high price and poor accessibility in many countries limit its use in many health care systems^4^. Furthermore, its fast reversal kinetics and complete reversal of neuromuscular blockade may result in unwanted patient movement during surgery^5,6^. Neostigmine, a cholinesterase inhibitor, is a safe, effective, and comparatively inexpensive alternative reversal agent^7,8^. The feasibility of neostigmine as a reversal agent for IONM during thyroid surgery was demonstrated in one previous observational study^9^. However, there is no strong evidence for using neostigmine to create a good condition for IONM in thyroid surgery.

The purpose of the present study was to investigate the effects of neostigmine on IONM during thyroid surgery compared with no use of reversal agents for IONM. We hypothesized that neostigmine would shorten the time to obtain good conditions for successful IONM, without increasing intraoperative bucking events.

## Methods

This prospective randomized controlled trial was approved by the Institutional Review Board of the Seoul Metropolitan Government Seoul National University Boramae Medical Center (no. 10-2021-12) and was registered on ClinicalTrials.gov (NCT04873531; https://register.clinicaltrials.gov/prs/app/action/ViewOrUnrelease?uid=U00026JX&ts=16&sid=S000AXJ8&cx=tqaqzt, 26 April 2021) before enrolment of subjects. Adult patients (≥ 19 years old) with American Society of Anesthesiology physical status I-III who were scheduled to undergo thyroid surgery with IONM were enrolled between May 2021 and August 2021. Patients who required neuromuscular blocking agents other than rocuronium or were scheduled to undergo endoscopic or robotic thyroid surgery were excluded from the study. Written informed consent was obtained from all patients.

### Randomization

Patients were randomly allocated in a 1:1 ratio to the neostigmine group (Group N) or the control group (Group C). Patients in Group N (n = 22) were administered 0.03 mg/kg neostigmine with 0.006 mg/kg glycopyrrolate after tracheal intubation, and those in Group C (n = 22) were administered the same volume of normal saline after tracheal intubation. Patients in Group C were administered neostigmine and glycopyrrolate after skin closure. An investigator who did not have a clinical role in this study generated the random allocation sequence using computer-generated block randomization (size: 6 blocks). The patient, operating surgeon, and surgical assistants were blinded to the allocation.

### Anesthesia

Upon patient arrival to the operating theater without premedication, standard monitoring was applied, including pulse oximetry, electrocardiography, and noninvasive blood pressure. Bispectral index monitoring was applied to obtain an adequate depth of general anesthesia with maintenance of a value of 40–60. Acceleromyography was applied at the adductor pollicis muscle and ulnar nerve of the left hand for evaluation of neuromuscular blockade. After administration of 30 mg lidocaine, induction and maintenance of anesthesia were performed with a continuous infusion of propofol and remifentanil using a target-controlled infusion system with Orchestra™ infusion pumps (Fresenius Vial, Brézins, France). After loss of consciousness, 0.6 mg/kg rocuronium was administered to relax the muscles for tracheal intubation.

The patient was positioned for thyroid surgery with a standard pillow beneath their neck and back prior to tracheal intubation to prevent tracheal tube displacement during positioning of the patient for surgery^10^. A nerve integrity monitor (NIM) standard reinforced EMG endotracheal tube (Medtronic, Jacksonville, FL, USA) with a 7.0 mm internal diameter for men and a 6.0 mm internal diameter for women was used for tracheal intubation. A single anesthesiologist (J-M.L.) performed tracheal intubation by direct laryngoscopy in all patients. The anesthesiologist aimed to place the midpoint of the EMG tube electrodes between both vocal cords while evaluating the modified Cormack-Lehane grade. After tube fixation, the anesthesiologist administered a mixture of 0.03 mg/kg neostigmine with 0.006 mg/kg glycopyrrolate to the patients in Group N or normal saline of the same volume to the patients in Group C. This process ensured the surgeon remained blinded to group allocation.

All setup and monitoring processes were performed in compliance with the standards outlined in the International Neural Monitoring Study Group guidelines^11^. The NIM response 3.0 system (Medtronic, Jacksonville, FL, USA) was used for nerve monitoring. The stimulation duration was set at 100 ms, the event threshold was set at 100 μV, and the stimulus current was set at 1 mA with a frequency of 4 Hz.

### Operative techniques

Operations were performed by a single surgeon (Y.J.C) who was blinded to the patient’s group allocation. A conventional low collar skin incision was made along the skin crease at the lower neck. After the isthmus was divided, the avascular plane between the cricothyroid muscle and the upper pole of the thyroid gland was exposed. After mobilizing the upper pole, EMG signals were recorded according to standardized IONM procedures^11^. During the operation, the surgeon checked whether the signal from the vagus nerve stimulation (V1) was successful. We defined the time of the initial V1 signal check as the time when the surgeon first tested the V1 signal after exposure of the carotid sheath, and the time of successful V1 stimulation as the time when a V1 signal > 100 μV was detected four or more times in succession under electrical stimulation currents of 1 mA at a frequency of 4 Hz. If successful V1 stimulation was not detected, the surgeon halted the operation and rechecked the V1 signal at 1-min intervals until successful V1 stimulation was detected. The time of the initial V1 check and successful V1 stimulation were recorded. After successful V1 stimulation, the surgeon continued the operation, which included confirmation of the RLN (R1) signal. The time and amplitudes of successful V1 and R1 stimulations were recorded. The train-of-four (TOF) ratio was recorded at the points of administration of rocuronium, tracheal intubation, administration of neostigmine or normal saline, and signal checks for V1 and R1. The surgeon suspended the operation if there was significant patient movement or bucking during surgery, and resumed procedures when the event was resolved. After the mass was completely removed, the surgeon checked the signals of the recurrent laryngeal nerve (R2) and the vagus nerve (V2) to recheck whether nerve impairment by surgery occurred. In patients who underwent total thyroidectomy, only the results of the lobe that was approached first were recorded.

### Outcomes

The primary outcome was the time from skin incision to successful V1. We defined a case with ‘time delay for V1’ as the case when V1 was not detected at the initial V1 check, and recorded the time between the initial V1 check to successful V1. The secondary outcomes included the occurrence of intraoperative bucking events and the mean EMG amplitudes of the V1, R1, R2, and V2 signals. The time when the anesthesia induction began was recorded as the time at which the observation started.

### Statistics

Quantitative variables of patient characteristics and outcome measures are presented as the mean ± standard deviation (SD). Categorical variables are presented as numbers with percentages. Depending on the distribution of variables according to the Kolmogorov–Smirnov test, continuous variables were compared using Student’s t test. Binary outcomes were compared using χ^2^ tests or Fisher’s exact tests. For the primary outcomes, the incidence of time delay was compared by Fisher’s exact test, and the time from skin incision to successful V1 stimulation was compared by Student’s t test between the two groups. For the secondary outcomes, the amplitudes of V1, R1, R2, and V2 were compared by Student’s t test and the incidence of the intraoperative bucking events was compared by Fisher’s exact test between the two groups. A *p* value less than 0.05 was considered statistically significant. Statistical analyses were performed using SPSS Statistics v. 26 software (IBM Corp., Armonk, NY, USA).

### Sample size calculation

One study reported that satisfactory qualitative nerve monitoring was achieved in 95% of patients after the administration of a combination of 2.5 mg neostigmine and 0.4 mg glycopyrrolate. In patients who received a placebo, satisfactory nerve monitoring was attained in only half of the cases^12^. We therefore assumed that the use of neostigmine and glycopyrrolate for reversal of neuromuscular blockade could prevent a delay in 95% or more patients and that no administration could cause a delay in approximately 50% of patients. To test this hypothesis, 19 patients per group were needed, with a type I error of 0.05, and 90% power. Considering a dropout rate of 15%, the sample size was calculated to be 44 patients.

## Results

### Patient characteristics

**Figure 1:**
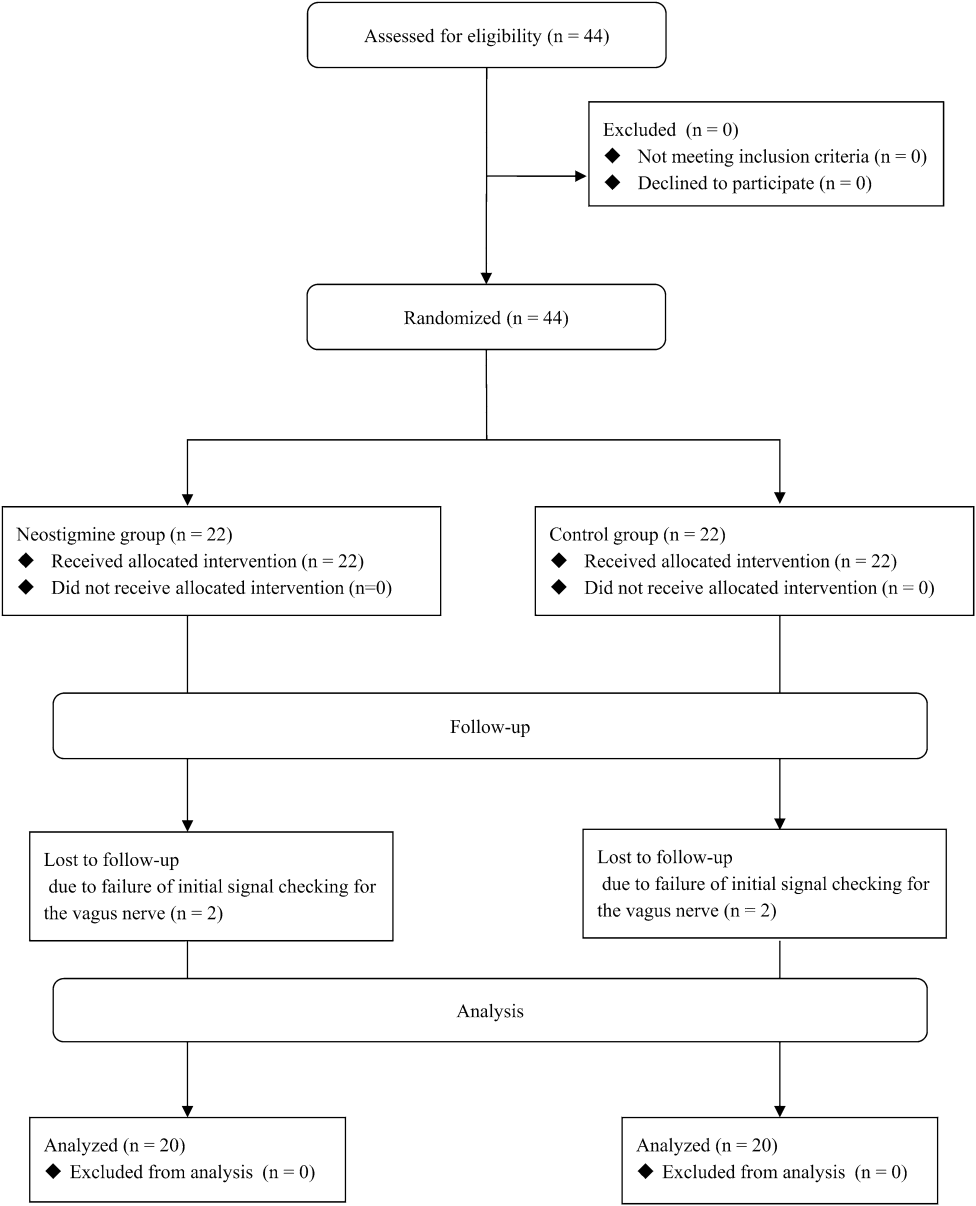
CONSORT flow diagram.

A total of 44 patients were enrolled in this study. Patient screening, enrolment, randomization, and analysis are shown in the CONSORT flow diagram in Fig. 1. The modified Cormack-Lehane grade was 1/2A/2B/3/4 in 2/18/16/6/0 among 42 patients in whom tracheal intubation was performed with a direct laryngoscope. Four patients who had no V1 signal after a significant amount of time were excluded from the analysis. Among them, two patients in Group N and one patient in Group C had no V1 or R1 signal but had laryngeal twitches. Postoperative laryngoscopic examination revealed normal vocal cord movement, suggesting that the absence of a V1 signal was due to technical failure. One patient in Group C had no V1 or R1 signal and no laryngeal twitch. Postoperative laryngoscopic examination revealed transient vocal cord palsy suggesting vagus nerve or RLN injury during upper pole manipulation or thyroid traction before the initial V1 signal check. Finally, 40 patients (20 patients in each group) were eligible for analysis. The clinical characteristics of the patients are summarized in Table 1. There were no significant differences between groups in terms of sex, age, body mass index, tumor size, operative extent, or pathological diagnosis. There were no adverse events, such as bradycardia or tachycardia, due to neostigmine-glycopyrrolate administration or any tissue injury by the involuntary intraoperative bucking movement of patients during the study. Additionally, no RLN injury occurred in any of the patients included in the analysis (Table 1).

**Table 1:** Patient characteristics.

Patient characteristics	Group N (N = 20)	Group C (N = 20)	*p* value
Gender (male:female)	6:14	3:17	0.256
Age (years)	56.6 ± 10.8	55.7 ± 12.1	0.850
Body mass index (kg/m^2^)	24.7 ± 4.1	23.8 ± 3.9	0.497
Tumor size in longest diameter (cm)	1.5 ± 1.4	1.3 ± 1.1	0.676
Extent of operation			0.999
**Lobectomy**	16 (80.0)	16 (80.0)	
Total thyroidectomy	4 (20.0)	4 (20.0)	
**Diagnosis**			0.711
Papillary thyroid carcinoma	16 (80.0)	14 (70.0)	
Follicular adenoma	3 (15.0)	3 (15.0)	
Nodular hyperplasia	1 (5.0)	1 (5.0)	
Hurthle cell carcinoma	0 (0.0)	1 (5.0)	
Hurthle cell adenoma	0 (0.0)	1 (5.0)	

All data are presented as mean ± standard deviation or as n (%), unless stated otherwise.

### Time measurements of anesthetic and operative procedures

Table 2 shows the duration of the anesthetic and operative procedures. The mean time from rocuronium injection to tracheal intubation was 3.5 ± 0.9 min for Group N and 3.8 ± 0.8 min for Group C (*p* = 0.265). The mean time from skin incision to initial V1 signal check was comparable between the two groups (15.4 ± 2.4 vs. 15.0 ± 2.5 min, *p* = 0.606). For all patients in Group N, V1 stimulation was successful on the initial attempt, whereas half of the patients in Group C (10/20) required multiple attempts before successful stimulation of V1 (*p* < 0.001), with a mean delay time of 11.2 ± 1.4 min between the initial attempt and successful V1 stimulation. As the primary outcome in our study, the mean time from skin incision to successful V1 stimulation was significantly shorter in Group N than in Group C (15.4 ± 2.4 vs. 19.9 ± 5.7 min, *p* = 0.003). The mean time from skin incision to R1 stimulation was also shorter in Group N than in Group C (17.1 ± 2.5 vs. 23.4 ± 6.5 min, *p* < 0.001). The mean time from skin incision to successful R2/V2 stimulation and total operative time were comparable between Group N and Group C (31.26 ± 6.7 vs. 34.6 ± 7.7 min, *p* = 0.139 and 48.6 ± 10.8 vs. 54.3 ± 16.3 min, *p* = 0.196).

**Table 2:** Time measurements of anesthetic and operative procedures.

Description	Group N (N = 20)	Group C (N = 20)	*p* value
Rocuronium to tracheal intubation, min	3.5 ± 0.9	3.8 ± 0.8	0.265
Tracheal intubation to neostigmine or placebo, min	3.5 ± 0.9	3.2 ± 1.1	0.401
Tracheal intubation to skin incision, min	9.8 ± 1.8	9.0 ± 1.6	0.167
Skin incision to initial V1 check, min	15.4 ± 2.4	15.0 ± 2.5	0.606
Time delay for V1, n	0	10	< 0.001
Time delay for V1, min	N/A	11.2 ± 1.4	N/A
Tracheal intubation to successful V1 check, min	25.2 ± 3.0	28.9 ± 5.6	0.013
Skin incision to successful V1, min	15.4 ± 2.4	19.9 ± 5.7	0.003
Skin incision to R1, min	17.1 ± 2.5	23.4 ± 6.5	< 0.001
Skin incision to R2/V2, min	31.2 ± 6.7	34.6 ± 7.7	0.139
Total operation time, min	48.6 ± 10.8	54.3 ± 16.3	0.196

All data are presented as mean ± standard deviation, unless stated otherwise.

*EBSLN* external branch of the superior laryngeal nerve, *V1* initial electromyography signal of the vagus nerve before surgical dissection, *R1* initial electromyography signal of the recurrent laryngeal nerve upon its initial identification, *R2* electromyography signal of the recurrent laryngeal nerve after thyroidectomy, *V2* electromyography signal of the vagus nerve after thyroidectomy.

### IONM quality and outcomes

IONM quality and outcomes are presented in Table 3. At the initial V1 signal check, the TOF counts were higher in Group N than in Group C (*p* < 0.001). The respective TOF counts were 0/4, 1/4, 2/4, 3/4, 4/4 in 1, 4, 4, 0, 11 patients in Group N; and 0/4, 1/4, 2/4, 3/4, 4/4 in 16, 3, 1, 0, 0 patients in Group C. At successful V1 stimulation, the TOF counts were significantly higher in Group N than in Group C (*p* < 0.001). The respective TOF counts were 0/4, 1/4, 2/4, 3/4, 4/4 in 1, 4, 4, 0, 11 patients in Group N and 0/4, 1/4, 2/4, 3/4, 4/4 in 10, 7, 3, 0, 0 patients in Group C. With R1 stimulation, the TOF counts were higher in Group N than in Group C (*p* = 0.019). For the secondary outcomes of our study, all patients in both groups except one in Group C had V1 amplitudes higher than 500 μV, and the mean V1 amplitudes were comparable between Groups N and C (962.2 ± 434.5 μV vs. 802.3 ± 382.7 μV, *p* = 0.225). All patients in both groups had R1 amplitudes higher than 500 μV, and the mean R1 amplitudes were comparable between Groups N and C (1240.0 ± 836.5 μV vs. 1023.4 ± 455.8 μV, *p* = 0.316). With R2 and V2 stimulation, TOF counts were not significantly different between groups. All patients in both groups had R2 and V2 amplitudes higher than 500 μV. Patients in Groups N and C had comparable mean R2 amplitudes (1186.8 ± 599.4 μV vs. 1098.5 ± 440.3 μV, *p* = 0.599) and V2 amplitudes (930.2 ± 433.3 μV vs. 893.3 ± 302.1 μV, *p* = 0.599). During the surgical procedure, bucking events were observed in one patient (5.0%) in Group N at the time of V1 stimulation and none in Group C, with no significant difference between the two groups (*p* = 0.311). The bucking movement in the patient in Group N subsided spontaneously within 1 min while the surgeon held the surgical procedures for a while.

**Table 3:** Intraoperative neuromonitoring quality and outcomes.

Description	Group N (N = 20)	Group C (N = 20)	*p* value
**Initial V1 check**			
TOFr (0/4, 1/4, 2/4, 3/4, 4/4)	1/4/4/0/11	16/3/1/0/0	< 0.001
TOFr, % for 4/4	50.4 ± 20.7	N/A	N/A
Amplitude of, μV (range)	962.2 ± 434.5 (515–2158)	826.6 ± 240.0 (565–1398)	0.369
**Successful V1**			
TOFr (0/4, 1/4, 2/4, 3/4, 4/4)	1/4/4/0/11	10/7/3/0/0	< 0.001
TOFr, % for 4/4	50.4 ± 20.7	N/A	N/A
Amplitude of, μV (range)	962.2 ± 434.5 (515–2158)	802.3 ± 382.7 (346–2124)	0.225
**R1**			
TOFr (0/4, 1/4, 2/4, 3/4, 4/4)	1/4/3/1/11	7/4/6/1/2	0.019
TOFr, % for 4/4	58.0 ± 19.2	19.2 ± 2.1	0.025
Amplitude, μV (range)	1240.0 ± 836.5 (573–4132)	1023.4 ± 455.8 (513–2043)	0.316
**R2 and V2**			
TOFr (0/4, 1/4, 2/4, 3/4, 4/4)	0/1/1/0/18	1/5/3/1/10	0.093
TOFr, % for 4/4	79.1 ± 25.8	58.3 ± 14.9	0.012
Amplitude of R2, μV (range)	1186.8 ± 599.4 (446–2840)	1098.5 ± 440.3 (516–1968)	0.599
Amplitude of V2, μV (range)	930.2 ± 433.3 (274–1829)	893.3 ± 302.1 (513–1695)	0.599
**Bucking events (%)**	1 (5.0)	0 (0.0)	> 0.999

All data are presented as mean ± standard deviation, unless stated otherwise.

*TOFr* train-of-four ratio, *V1* initial electromyography signal of the vagus nerve before surgical dissection, *RLN* recurrent laryngeal nerve, *R1* initial electromyography signal of the recurrent laryngeal nerve at its initial identification, *R2* electromyography signal of the recurrent laryngeal nerve after thyroidectomy, *V2* electromyography signal of the vagus nerve after thyroidectomy.

## Discussion

The results of the present study show that compared with placebo, the use of neostigmine for IONM provided a faster time to successful IONM. The mean time from skin incision to successful V1 stimulation was 15.4 ± 2.4 min for Group N and 19.9 ± 5.7 min for Group C (*p* = 0.003) in this study. In a previous study, Gunes et al.^13^ reported that in patients for whom no reversal agents were used the mean time from the point of initiation of surgery to the point of full recovery of laryngeal EMG was 50 min. Our center recently reported that the mean time from neostigmine administration to stimulation of the external branch of the superior laryngeal nerve was 21.0 ± 4.5 min^9^. Previous studies either reported results of IONM when no reversal agents were used or results of IONM when neostigmine was used as a reversal agent^9,13^. However, there has been a lack of comparison studies on this issue. To the best of our knowledge, this is the first randomized controlled trial to compare the IONM quality and the time to obtain adequate conditions for IONM between patients who were administered neostigmine as a reversal agent before the initiation of surgery and those who were not.

There are currently no clear guidelines on the management of neuromuscular blockade for IONM during thyroid surgery. Various strategies have been suggested for the optimal management of neuromuscular blockade. Some studies have focused on manipulating NMBAs, such as using relaxant-free anesthesia or lower doses of NMBAs^14,15^. However, it has been shown that avoiding NMBAs cannot improve tracheal intubation conditions and can increase the risk of difficult tracheal intubation^16^. Considering the potentially serious consequences of difficult tracheal intubation, good IONM conditions should never be prioritized over good tracheal intubation conditions. Therefore, we used rocuronium as an NMBA with a reversal agent when IONM was planned. Previous studies have reported sugammadex after rocuronium use is useful for IONM^13,17,18^. While some studies suggest a dose of 2 mg/kg sugammadex for reversal of neuromuscular blockade prior to IONM^13,17^, one study comparing 1 mg/kg and 2 mg/kg sugammadex showed that a dose of 1 mg/kg sugammadex induced fewer intraoperative bucking events than a 2 mg/kg dose, while still providing comparable IONM quality^5^. This demonstrates that the goal of administering NMBAs is not complete recovery from neuromuscular blockade, but just enough reversal so that IONM is possible while still providing good anesthetic and surgical conditions. In fact, when successful V1 stimulation was detected in Group C, the TOF counts were all below 3/4, but high-quality IONM was still possible. Because the threshold for the reversal of neuromuscular blockade is different for each muscle and nerve of the body, TOF monitoring applied at the adductor pollicis muscle may not have been able to accurately reflect reversal of muscles innervated with the vagus nerve or the RLN. This is in line with previous studies that have suggested that TOF monitoring may be of little value in IONM^9,19,20^. Therefore, we believed that reversal agents less potent than sugammadex, such as neostigmine, could be an alternative reversal agent for qualified IONM.

A previous observational study presented the use of neostigmine as a possible reversal agent for IONM^9^. Neostigmine is a commonly used reversal agent because it is safe, effective, inexpensive, and readily available^21^. The present study showed that the use of neostigmine after tracheal intubation did not cause a time delay until IONM was possible and provided adequate IONM quality without significantly increasing the incidence of intraoperative bucking movement during thyroid surgery. Now, it has been established that the use of neostigmine is beneficial for IONM during thyroid surgery in comparison with no use of reversal agents. Further studies comparing neostigmine and sugammadex are needed to determine the optimal strategy for IONM in thyroid surgery.

The optimal time to administer reversal agents is also unknown. Many studies have reported the administration of reversal agents directly before IONM^13,17,18,22^. One study noted that when sugammadex was administered directly after tracheal intubation, up to 35% of patients experienced intraoperative bucking events, more than 60% of which occurred during skin incision and flap dissection^5^. Therefore, delayed administration of sugammadex may be beneficial, especially after strong surgical stimuli, such as skin incision or flap dissection. However, delayed administration of neostigmine may not have the same effect as sugammadex because neostigmine results in slower and less complete reversal. We administered neostigmine after tracheal intubation because we assumed that the effect of neostigmine might be maximized when administered after tracheal intubation. Neostigmine works by blocking acetylcholinesterase activity, resulting in an increase of acetylcholine levels in the body. Its peak effect occurs in 10 min and duration of action is 20–30 min^23^. Considering the pharmacokinetics of neostigmine and the relatively short time from tracheal intubation to the initial V1 check (approximately 25 min) in our routine clinical practice, we planned to administer neostigmine after tracheal intubation even though it takes a longer time for the TOF ratio to recover if neostigmine is administered at the point of deep neuromuscular blockade^24^. In our experience, a high TOF ratio was not necessarily required for successful IONM during thyroid surgery^5^. One study in which neostigmine was given 30 min after NMBA administration for the induction of anesthesia reported that the IONM quality was significantly improved compared to that when no reversal agent was used^12^. In our study, the mean operative time was 48.6 ± 10.8 min and 54.3 ± 16.3 min in Groups N and C, respectively, and the mean time from rocuronium administration to neostigmine or placebo administration was approximately 7 min in all patients in both groups. The administration of reversal agents 30 min after anesthesia induction is not suitable for centers with short operative times. A previous IONM study, in which reversal agents were administered just after tracheal intubation, reported that only two out of 50 patients (4%) experienced intraoperative bucking events^9^. In the present study, the same timeline as the previous study was used for neostigmine administration in Group N, and one out of 20 patients (5%) in the group experienced intraoperative bucking events, which was lower than other reports in which sugammadex was administered^5,6^. Nevertheless, more research is needed to establish the optimal timing for the administration of reversal agents.

In this study, we defined four or more consecutive V1 signals > 100 μV responding to electrical stimulation currents as successful V1 stimulation. To the best of our knowledge, there is no clear reference on the definition of successful V1 stimulation. Therefore, we defined successful V1 stimulation by partially using the concept of TOF^25^. Four twitches to four stimulations means that approximately 25–30% recovery from neuromuscular blockade. Of course, this is not enough to recover self-respiration, but we believe that a nearly full recovery from neuromuscular blockade (TOF ratio > 0.7 or 0.9) is not required to obtain adequate conditions for IONM. In our protocol, we showed that the amplitude of the signal of V1 was sufficiently high (> 500 μV in 39 patients of both groups) when four or more consecutive twitches were detected upon V1 stimulation, even though the TOF count at the adductor pollicis muscle was 3 or less in 9/20 patients in Group N and 20/20 patients in Group C.

The question of whether sufficient neostigmine-induced reversal to create a clinically adequate condition for IONM happens as a sudden event or as a gradual process has not been previously addressed. In the present study, we defined four or more consecutive twitches responding to electrical stimulation as successful V1, and we rechecked the signal repeatedly in 1-min intervals until a successful V1 signal was produced for patients with unsuccessful initial V1 stimulation. Although we continuously checked the V1 signal to electrical stimulation at 1-min intervals in this study, the surgeon observed that successful V1 stimulation appeared rather abruptly. In other words, even if the recovery progress from neuromuscular blockade occurs gradually^26^, sudden successful V1 stimulation can be achieved in terms of the clinical condition for IONM, e.g., abrupt successful V1 stimulation 1 min after no or only one twitch. This might suggest that the neuromuscular blockade recovery needed for adequate IONM by reversal agents occurs in a narrow period of time. Even if reversal agents are not given, V1 should be checked at least every minute to minimize unnecessary loss of time in surgery.

There are some limitations to this study. First, factors other than the NMBAs and reversal agents may have affected the EMG results and were not controlled in the analysis. EMG amplitudes evoked by nerve stimulation vary from stimulation to stimulation and from patient to patient for multiple reasons including malposition of the endotracheal tube, manipulation of the gland or trachea during nerve stimulation, the volume of fluid in the surgical field, and nerve ensheathment within the fascia that may obstruct probe-to-nerve contact. Although we could not control for each of these points, much effort was put into creating a controlled environment for all surgical procedures. We also believe that this limitation was overcome to some extent by our randomized study design, despite the small sample size. Second, although we previously proposed using neostigmine as an alternative to sugammadex for IONM in thyroid surgeries, we did not compare neostigmine to sugammadex in the present study. This was because we aimed to first establish the feasibility of using neostigmine in comparison with the absence of reversal agents. We expect to see future studies that investigate the effects of neostigmine versus sugammadex as reversal agents for IONM during thyroid surgeries. Last, surgery was performed by a specialized and experienced endocrine surgeon, and thus, the operation times were relatively short. Therefore, the results of this study may not be generally applicable, and much caution is needed in the interpretation of the results. Nevertheless, the strength of this study was its randomization of patients, which prevented potential selection bias. We observed significant differences in the results between the two groups despite a small sample size.

In conclusion, the administration of neostigmine immediately after tracheal intubation successfully reversed the neuromuscular blockade induced by rocuronium without an increase in intraoperative bucking events. Therefore, neostigmine administration immediately after tracheal intubation may be useful to reverse neuromuscular blockade for successful IONM in thyroid surgeries.

## Data Availability

The datasets generated during and/or analyzed during the current study are available from the corresponding author on reasonable request.
